# Prevalence of intestinal parasitic infections in certified food-handlers working in food establishments in the City of Nairobi, Kenya

**DOI:** 10.1016/S1674-8301(12)60016-5

**Published:** 2012-03

**Authors:** Paul Kamau, Penina Aloo-Obudho, Ephantus Kabiru, Kepha Ombacho, Bernard Langat, Obadiah Mucheru, Laban Ireri

**Affiliations:** a Department of Zoological Sciences, Kenyatta University, Nairobi, Kenya;; b Department of Pathology, Kenyatta University, Nairobi, Kenya;; c Ministry of Public Health and Sanitation, Nairobi, Kenya;; d Institute of Tropical Medicine and Infectious Diseases, Jomo Kenyatta University of Agriculture and Technology, Nairobi, Kenya;; e Division of Vector Borne and Neglected Tropical Diseases, Embu, Kenya.

**Keywords:** parasites, food-handlers, medical certificate, food establishments

## Abstract

Most intestinal parasites are cosmopolitan with the highest prevalence in the tropics and subtopics. Rural-to-urban migration rapidly increases the number of food eating places in towns and their environs. Some of these eating estabishments have poor sanitation and are overcrowded, facilitating disease transmission, especially through food-handling. Our investigations in Nairobi, therefore, were set to determine the presence of intestinal parasites in food-handlers with valid medical certificates. Direct and concentrated stool processing techniques were used. Chisquare test and ANOVA were used for data analysis. The parasites *Ascaris lumbricoides, Entamoeba histolytica* and *Giardia lamblia* were observed in certified food-handlers. Significant difference was found in parasite frequency by eating classes and gender (χ^2^ = 9.49, *P* = 0.73), (*F* = 1.495, *P* = 0.297), but not in parasite occurrence between age brackets (χ^2^ = 6.99, *P* = 0.039). The six-month medical certificate validity period may contribute significantly to the presence of intestinal parasites in certified food-handlers.

## INTRODUCTION

Parasitic infections, including infectious diseases, develop mainly in poor people and vary with age in the poorest countries of the world. The parasites primarily infest the small intestines and colon in man and other animals[1]. The morbidity associated with these is strongly related to the parasite burden[2],[3]. Parasitic diseases thus contribute to inequalities that exist both within and between societies[4]. The present distribution of intestinal parasitic diseases reflects the success of hygiene and control measures in the more developed parts of the world, rather than any clear geographical or climatic restriction[5]. In many communities, 15% of the infected population harbours more than 60% of the worms. Food establishments play a notable role in the food industry but they may, sometimes, be sources of parasitic and other infections. Human beings acquire intestinal parasitic infections through several routes, including faecal-oral and skin penetration, and it causes untold suffering[6]-[8]. Food-handlers may serve as the transmission agents and reservoirs of infection[9]-[13]. Limited health education in some food-handlers and lack of personal hygiene may be the causes of these infections. They can get infected with parasites before the expiration of their medical certificates and may pass on infections to uninfected colleagues and clients. The aim of this study was to investigate whether food-handlers with valid medical certificates acquire intestinal parasitic infections before the six-month expiration period and to unravel remedial measures.

## MATERIALS AND METHODS

### Study area

The study was carried out in Nairobi, the capital City of Kenya. It lies at an attitude of 36°50′ East and latitude 1°17′ South. It is located 480 km inland northwest of the Indian Ocean, 140 km south of the equator and has an area of 684 km^2^, and 312 food-handlers were sampled in 48, 17 and 5 food eateries in low, middle and high classes, respectively. Except during the months of July and August, which are distinctly cool, the climate is warm[14].

Randomized stool sampling from various food outlet categories was done in the Nairobi Central Business District and its environs in the ratios of 131:105:76 for low, middle and high class, respectively, to make a total of 312 food-handlers. The sample size was determined using the formula:

N = 
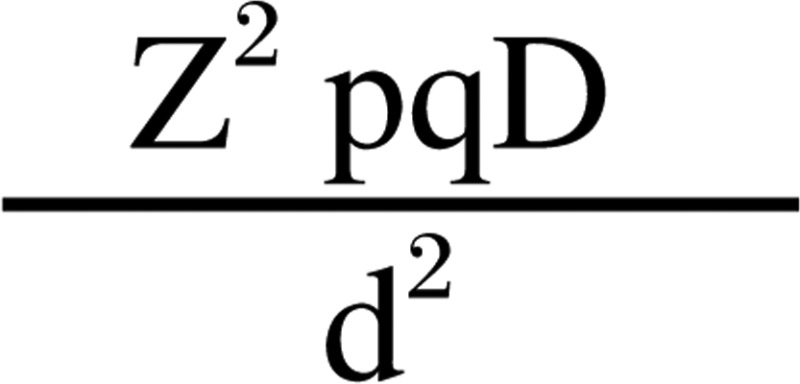
[15]. Sampling was carried out weekly for a period of 12 months in 48, 17 and 5 eating places serving the low, middle and high-classes, respectively. Stool samples were processed using direct and concentration techniques and examined under a microscope for intestinal parasites. Two stool samples per food handler were examined within 7 d.

### Subjects

The subjects enrolled were food-handlers with valid medical certificates who worked in Nairobi food outlets. Because food-handlers working in food outlets, by July 2006, numbered above 108,000, according to the Public Health Department in the City Council of Nairobi, the total sample size reasonably considered was 312. The inclusion criteria was having valid medical certificates and providing informed consent for the study. Those who declined to participate or did not have valid certificates were excluded. Food eating places were categorized into three classes, i.e., the low, middle and high, and represented food kiosks, middle-level eating-houses and hotels, respectively. In kiosks, food-handlers are usually illiterate or semi-literate with minimal health education in personal hygiene. Those in mid-level eating houses are usually semi-literate or literate while those in hotels are literate and well-trained in catering. A rich social-cultural diversity contributed to the selection of Nairobi City as a suitable study area. Systematic sampling of food establishments was done and every third eatery was sampled. To obtain the required sample size per eatery, convenience sampling was done. This depended on the cooperation of the respective managers and food handlers with valid medical certificates. If cooperation was unsuccessful, the next eatery was sampled. Because kiosks greatly outnumber all the other eating outlets in the City of Nairobi, while hotels are the least numerous, probability proportional to size sampling was carried out. Food-handlers working in the low, middle and high eateries were sampled at the ratios of 5:3:2, respectively, to make a total sample size of 312. The low, middle and high-class food outlets were represented by 131, 105 and 76 food-handlers, respectively. The sampling was done from Mondays through Thursdays during the course of the study. On the first visit, the researchers introduced themselves to the managers and the food-handlers. Fresh stool samples were collected from food-handlers who had valid medical certificates and had provided informed consent to participate in the study. The stool samples were then submitted to Rhodes and Casino Health Facility Laboratories for examination. This took place on Tuesdays to Fridays for 4 months. The procedure was conducted for low, middle and high class food outlets in the study area. Classification depended on the type of licenses given to the food establishments.

Prior to data collection, the protocol was reviewed and approved by the Kenya Ministry of Education, Science and Technology, City Council of Nairobi as well as Kenyatta University Institutional Review Board. A questionnaire was developed and used to obtain pertinent data from the food-handlers who provided informed consent for this study.

### Direct microscopy technique

Direct stool examination was carried out using techniques previously described[17]. Briefly, two wet preparations of fresh stool from the same food-handler were made as follows: a drop of fresh normal saline was placed on one end of a microscopic glass slide and a drop of Lugol's iodine on the other end. The proper amount of stool specimen (0.25 mg) was picked with an applicator stick and emulsified with the formal saline on one end of a glass slide; a same-size stool sample was treated in the same way with the Lugol's iodine on the opposite end of the same slide. The two preparations were then covered with glass cover slips (22 mm×22 mm) and examined under an ordinary light microscope for the presence of any parasites. The different intestinal parasites species identified were recorded with respect to eatery class, validity period of the medical certificate, gender, age, and educational level.

### Formal ether concentration technique

The concentration technique was carried-out using procedures previously described[17], except, briefly, 3 g of fresh stool sample were emulsified in 7 mL of formal saline. The resulting suspension was filtered through three layers of wet cotton gauze in a funnel into a centrifuge tube and 3 mL of diethyl ether was added. The centrifuge tube was corked, shaken vigorously and then centrifuged at 1000 *g* to 2500 *g* for 3-5 min. The plug was dislodged with an applicator stick and the supernatant poured off. Two wet preparations were made out of the deposit after it was slightly shaken, covered using a glass cover slip (22 mm×22 mm) and examined for the presence of parasites as above.

### Statistical analysis

Fisher's exact test was used to analyze for any significant difference in parasite frequencies in certified food-handlers by low, middle and high eating class categories. Parasite relationship to age strata, education and gender was determined.

## RESULTS

*Entamoeba histolytica (E. histolytica)* cysts and/or trophozoites were observed in 29, 9 and 1 certified food handlers in low, middle and high-class eating places, respectively. There were also 3, 1, and 0 *Giardia lamblic (G. lamblia)* cysts and 5, 1 and 0 *A. lumbricoides* ova in the low, middle and high-class samples, respectively. Out of the 312 food-handlers sampled, 49 or 15.7% had different species of parasites and 6 of the 49 or 1.9% had *Ascaris lumbricoides* (*A. lumbricoides*). Generally, parasites were more frequently seen in males than females in the different eating place classes sampled (***Table 1***). *E. histolytica* were found in 39 (12.5%) of the subjects while *G. lamblia* was found in 4 (1.3%). The majority of the food-handlers, 263 (84.3%), did not present with such infections. Other types of intestinal parasites were not detected in the stool samples. No significant difference was found in the infection frequency by the three eatery classes (*P* < 0.05).

**Table 1:** Number and distribution of *E. histolytica, A. lumbricoides* and *G. lamblia* in the different classes of food establishments and gender

Class	Gender	Parasite		
		*E. h*	*A. 1*	*G. 1*
Low	Male	18(5.7)*	2(0.64)	2(0.64)
	Female	11(3.53)	3(0.96)	1(0.32)
Middle	Male	4(1.28)	1(0.32)	1(0.32)
	Female	5(1.6)	0	0
High	Male	1(0.32)	0	0
	Female	0	0	0

*Relative to the total samples examined, *P* < 0.05 (Fisher's exact test). E.h: *Entamoeba histolytica*; A.l: *Ascaris lumbrico*; G.l: *Giardia lamblia*.

[*n*(%), *n* = 376]

**Fig. 1:**
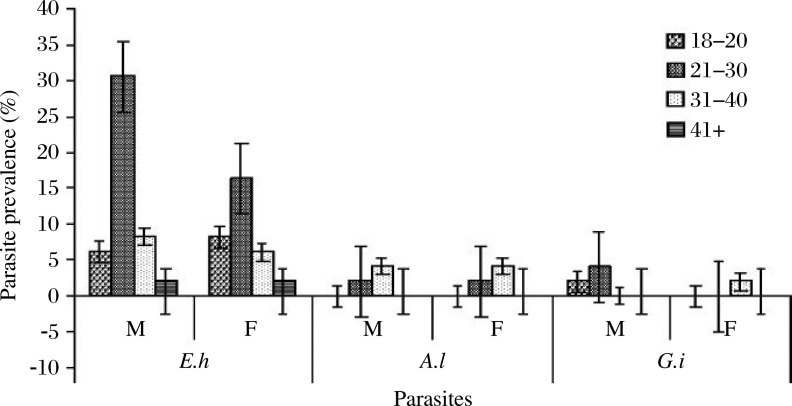
Parasite frequencies in certified food-handlers in four different age strata. E.h: *Entamoeba histolytica*, A.l: *Ascaris lumbricoides*, G.l: *Giardia lamblia*. M: male, F: female. *P* = 0.039, χ^2^ = 6.99 (Significance *P* < 0.05).

Regarding education levels, primary education was found in 68 (21.8%), secondary in 214 (68.6%), and university in 30 (9.6%) of the food-handlers sampled. Similarly, education levels and gender of the food-handlers had no significant correlation with parasite infection rates [education level (*P* = 0.976), gender (*P* = 0.772)]. The parasite occurrence was mainly noted in those in the age bracket of 21-30 y, where males were found harbouring the highest percentage of the parasites. The parasite occurrence was significantly different between age brackets (*P* = 0.032, ***Fig. 1***). The parasites were predominantly present during the final months of the certificate's validity period (***Table 2***).

**Table 2:** Frequency in counts of *E. histolytica, A. lumbricoides* and *G. lamblia* in the samples examined in the five month period in the three classes of food establishments

Class	Parasite	Duration (month)*				
		1^st^	2^nd^	3^rd^	4^th^	5^th^
Low	*E. histolytica*	0	1(2.04)	9(18.37)	9(18.37)	10(20.4) **
	*A. lumbricoides*	1(2.04)	1(2.04)	1(2.04)	1(2.04)	2(4.1)
	*G. lamblia*	0	0	2(4.1)	1(2.04)	1(2.04)
Middle	*E. histolytica*	0	1(2.04)	2(4.1)	2(4.1)	4(8.16)
	*A. lumbricoides*	0	0	0	1(2.04)	0
	*G. lamblia*	0	0	0	1(2.04)	0
High	*E. histolytica*	0	1(2.04)	0	0	0
	*A. lumbricoides*	0	0	0	0	0
	*G. lamblia*	0	0	0	0	0

*Duration = the number of months after the last medical examination

**Figures in parentheses represent percentage positive in relation to the total parasites encountered *(P* < 0.05, Fisher's exact test).

[*n*(%), *n* = 49]

## DISCUSSION

The findings from this study showed the intestinal parasites *E. histolytica, G. lamblia* and *A. lumbricoides* were found in some medically certified food-handlers with valid certificates. This could have been, in part, a result of the lengthy six-month validation period, a lack of basic food safety and health requirements, and inadequate personal hygiene. Food-handlers who had parasites were evenly distributed in relation to categories of eating places and gender. The positive cases were most commonly seen in the 21-30 y age bracket and least seen in the over 40 y bracket, depicting the active ages in these food outlets. The parasite *E. histolytica* was more prevalent in low-class (29; 9.29%) food outlets than *G. lamblia* and *A. lumbricoides* and were all less prevalent in high-class hotels, probably revealing the effect of hygiene practices. However, there was no significant (*P* > 0.05) statistical difference in intensity amongst food-handlers in all the food outlets. The parasites showed an upward trend, especially *E. histolytica*, as the period of the validity period decreased, possibly due to the incubation period of the undetectable number of the parasites, re-infection or both.

This study is concordant to studies by several other researchers who had shown that food-handlers could be good sources of parasitical intestinal infections. Andargie *et al*.[18] found that 29.1% of food-handlers were infected with intestinal parasites in Northwest Ethiopia. Additionally, Al-Lahham, *et. al*.[19] conducted a similar study in Jordan where the frequency of infection by intestinal protozoa was 30.2%, which is far much higher than in the just concluded study which stood at 13.8%. The reason could probably be due to dissimilar socio-economic status and environmental conditions associated with the two diverse populations. Also, these people are from poor families lacking proper housing, safe water supplies and hygienic waste disposal systems[20]. In Malaysia, about 15% of food-borne disease outbreak are the result of contamination by food-handlers[21]. Infected food-handlers have been implicated as vehicles in parasitic transmission, resulting in outbreaks in food eateries with poor sanitation posing risks to travellers[22],[23]. In Bahir Dar town in Ethiopia, 6.5% of food-handlers had protozoa and helminthic infection[24].

Although education levels could have influenced the pattern of infection, not all of the participants had access to formal health education on food safety[11]. Those who had access to primary school education were 21.8%, secondary education, 68.6% and only 9.6% had university education and were distributed among the food establishments. Education level in some East African countries such as Ethiopia, are lower than in Kenya[25]. Many food-handlers did not seem to be aware of basic safety and health requirements to work with food and food products. A mere 14% conceded having undergone an intensive food safety course. There was, however, no statistical significance (*P* > 0.05) between infection and education of the respondents. Studies by Babiker *et al*.[26] found that the infectivity of intestinal helminthes in Sudanese food-handlers was 2.7%, which compares to the findings of the present study where 1.92% of the food-handlers had helminthic infections. The findings of this study were of particular significance since infected food-handlers may be at risk of developing illness themselves, and may be a threat to the health of their clients[27]. Outbreaks of food borne diseases including those of intestinal infections periodically occur on virtually every continent, illustrating that unsafe food is a worldwide public health problem[28].

Although we expected that some seasonal variation in transmission of intestinal helminthes may feature here, the present study failed to detect any significant variation between occurrences of the infection in different months. This is in agreement with a similar study done in Khartoum by Babiker *et al*.[26], but contradicts a study done in Nigeria by Nzeako *et al*.[29] who reported seasonality of infection with intestinal helminthes. While access to safe and nutritious food is essential for life and is indeed the foundation for health, food markets, the sole suppliers of these food to eating places, they have unfortunately contributed significantly to the spread of a number of emerging disease agents[30]. The food eating places have in turn spread some of these infections to their clients through unhygienic food handling practices.

In conclusion, the prevalence of intestinal parasites may have been largely due to poor personal hygiene practices, the lengthy six months medical certificate validity period and environmental sanitation, lack of safe water, discrepancy in socioeconomic status, ignorance of health-promotion practices, and impoverished health services which should be addressed promptly. Food-handlers with valid medical certificates and working in all classes of food establishments get infected by intestinal parasitic agents including *E. histolytica, G. lamblia, A. lumbricoides* among others during the six month validity period. *E. histolytica, G. lamblia* and *A. lumbricoides* infections implied that these parasites were able to infect food-handlers in any type of food outlet with the same intensity, revealing important insights into possible transmission of other infectious agents if public health measures are compromised.

Food borne diseases surely have major economic impacts on individuals, food businesses and even countries and, therefore, large proportions of income may be lost by individuals due to reduced productivity and expenditures on medical care[28]. Mult-sectoral approach to food safety at all levels cannot be overemphasized[31]. We therefore recommend that all medically certified food-handlers have regular screenings, are supplied with antiamoebic and antihelminthic drugs at regular intervals, and/or are encouraged to participate in public health education programs. Food markets offering an array of street-vended foods, which are important sources of nutritious ready-to-eat foods that are accessible and affordable to even the lowest income earners of the community, should also be targeted for future studies. Similar studies are needed to establish whether Chapter 254 (Regulation 15) of the laws of Kenya governing certification of food-handlers should be reviewed.
